# Nanosilica and Polyacrylate/Nanosilica: A Comparative Study of Acute Toxicity

**DOI:** 10.1155/2016/9353275

**Published:** 2016-02-15

**Authors:** Ying-Mei Niu, Xiao-Li Zhu, Bing Chang, Zhao-Hui Tong, Wen Cao, Pei-Huan Qiao, Lin-Yuan Zhang, Jing Zhao, Yu-Guo Song

**Affiliations:** ^1^Occupational Disease and Toxicology Department, Beijing Chao-Yang Hospital, Capital Medical University, Beijing 100020, China; ^2^Department of Toxicology, National Institute for Occupational Health and Poison Control, Chinese Center for Disease Control and Prevention, Beijing 100050, China; ^3^Department of Respiratory and Critical Care Medicine, Beijing Institute of Respiratory Medicine, Beijing Chao-Yang Hospital, Capital Medical University, Beijing 100020, China; ^4^Department of Ultrasonography, Beijing Chao-Yang Hospital, Capital Medical University, Beijing 100020, China

## Abstract

We compared the acute toxicity of nanosilica and polyacrylate/nanosilica instillation in Wistar rats (*n* = 60). Exposure to nanosilica and polyacrylate/nanosilica showed a 30% mortality rate. When compared with saline-treated rats, animals in both exposure groups exhibited a significant reduction of PO_2_ (*P* < 0.05) at both 24 and 72 hr. after exposure. Both exposure groups exhibited a significant reduction of neutrophils in arterial blood compared to saline controls (*P* < 0.05) 24 hr. after exposure. The levels of blood ALT and LDH in exposed groups were found to be significantly increased (*P* < 0.05) 24 hr. following exposure. The exposed groups exhibited various degrees of pleural effusion and pericardial effusion. Our findings indicated respiratory exposure to polyacrylate/nanosilica and nanosilica is likely to cause multiple organ toxicity.

## 1. Introduction

In the past 20 years, the industrial and medical use of nanoparticles has expanded rapidly. Nanosilica is one of the most popular nanomaterials that are produced on an industrial scale as additives to cosmetics, drugs, printer toners, varnishes, and food. In addition, nanosilica is being developed in biomedical and biotechnological applications such as cancer therapy, DNA transfection, drug delivery, and enzyme immobilization [1–5]. More information on the impact of nanosilica on basic biology, medicine, and agronanoproducts can be found in a recent review published by Barik et al. [6].

With the growing commercialization of nanotechnology products, human exposure to nanosilica has inevitably increased. Many aspects such as sizes and surface areas of these nanomaterials have raised concerns about safety of ecological environment and human health [7–9]. Nanosilica might lead to multiple organ damage. Inhalation of nanosilica causes pulmonary inflammation, myocardial ischemic damage, and increase in fibrinogen concentration and blood viscosity [10]. Nanosilica exposure also results in DNA damage [11], size-dependent hydroxyl radicals generation [12], and lung fibrogenesis in rats [13]. Nanosilica could be preferentially distributed in liver, leading to liver injury [14, 15]. Nevertheless, wide range of toxicity and underlying mechanism have been reported including cellular nucleoplasmic protein aggregates [11], metabonomics [16], and oxidative stress and apoptosis [17, 18].

As a novel composite material, polyacrylate/nanosilica is a polymer of polyacrylate and nanosilica. Polyacrylate/nanosilica possesses both organic and inorganic particles at the nanometer size with the potential to be extensively used in multiple applications such as plastics, rubbers, and coatings [19–21]. A previous clinical trial reported by our group on polyacrylate/nanosilica showed increased worker mortality among those exposed to polyacrylate/nanosilica [22]. Pleural effusions, pulmonary fibrosis, and hypoxaemia were found in those workers, suggesting that polyacrylate/nanosilica as a material may require extra safeguards in an occupational setting [22]. However, the toxicological data on polyacrylate/nanosilica is scarce, illustrating the urgent need for more in-depth research prior to widespread industrial usage.

The research conducted in this study compared the acute toxicity between nanosilica and polyacrylate/nanosilica* in vivo* as an attempt to better understand the health effects associated with the two materials, especially about the novel composite material polyacrylate/nanosilica.

## 2. Materials and Methods

### 2.1. Animals and Materials

Specific pathogen-free (SPF) male Wistar rats (*n* = 60, 8 weeks old, 220 ± 10 g each, Beijing Weitong Lihua Experimental Animal Technology Co., Ltd.) were exposed via intratracheal instillation to either 0.9% saline (Tianjin Baxter Healthcare Ltd., national license medical number: H10983046); 20 nm silica (Department of Materials Science, Shanghai Fudan University); or 20 nm polyacrylate/nanosilica composite emulsion (Department of Materials Science, Shanghai Fudan University).

### 2.2. Nanocomplex Preparation

The nanoparticles were characterized by Tecnal G2 20S-TWIN TEM (FEI) in Key Laboratory of Standardization and Measurement for Nanotechnology, Chinese Academy of Sciences. Prior to exposure, animals were anesthetized using ethyl ether (Tianjin Jindong Tianzheng Fine Chemical Reagent Factory). An intraperitoneal (IP) injection of 50 mg/kg body weight of pentobarbital sodium was carried out (Beijing Chemical Reagents Company, import from German, subpackage, batch number: 020919) before blood sample collection. In preparation for instillation, particles were vortexed using a Vortex3000 vortex oscillator (Wiggens).

### 2.3. Clinical Characterization

Blood gases were analyzed using GEM Premier 3000 blood gas analyzer (American Experimental Instrument Co. Ltd.). Blood white blood cell count (WBC), neutrophil, and monocyte count were measured with XE2100 automatic blood analyzer (Sysmex Corporation). Serum alanine aminotransferase (ALT) and lactate dehydrogenase (LDH) were measured with AU2700 automatic biochemical analyzer (Olympus). In addition, S2000 color ultrasonic diagnostic instrument (SIEMENS, linear array probe 9L4) was used to monitor pulmonary injuries of the animals.

Rats were housed in a SPF animal room in Chinese Center for Disease Control and Prevention for 1 week. Animals were divided into 6 groups by the random number table method (*n* = 10 per group): (1) intratracheal instillation with 0.9% saline 24 hrs observation group, (2) intratracheal instillation with 0.9% saline 72 hrs observation group, (3) intratracheal instillation with nanosilica composite emulsion 24 hrs observation group, (4) intratracheal instillation with nanosilica composite emulsion 72 hrs observation group, (5) intratracheal instillation with polyacrylate/nanosilica composite emulsion 24 hrs observation group, and (6) intratracheal instillation with polyacrylate/nanosilica composite emulsion 72 hrs observation group. Rats in each group were weighed and then anesthetized with ethyl ether. Prior to instillation, nanosilica or polyacrylate/nanosilica composite emulsions were agitated via oscillation for 30 min. Rats were then exposed to about 0.5 mL of nanosilica, or polyacrylate/nanosilica composite emulsion at a dose of 102.4 mg/kg body weight while control group rats were exposed to 0.5 mL 0.9% saline. The experiments were conducted in accordance with the guidelines of Capital Medical University for the care and use of animals, with approval of the animal ethical committee of Capital Medical University.

At 24 and 72 hrs postexposure to nanosilica, polyacrylate/nanosilica mix, or saline, arterial blood was collected from the abdominal aorta for analyses, from which 3 mL was used to measure pH, PO_2_, and PCO_2_ levels, and 6 mL was used to quantify WBC, neutrophils, and monocytes, as well as alanine aminotransferase (ALT) and lactate dehydrogenase (LDH).

Two animals from each treatment were randomly selected to undergo ultrasound imaging. Animals were anesthetized via IP injection with 50 mg/kg pentobarbital sodium and prepped for pleural effusion and pericardial effusion. The ultrasound examinations were conducted 120 hrs. (5 days) following exposure.

### 2.4. Statistical Analysis

SPSS 16.0 statistical package was used to conduct statistical analysis. Measurement data was expressed as mean ± standard deviation. One-way analysis of variance was used to determine the significance of the difference among the group means. An alpha level of *P* < 0.05 was used to determine significance.

## 3. Results

### 3.1. Characterization of Nanoparticles

**Figure 1:**
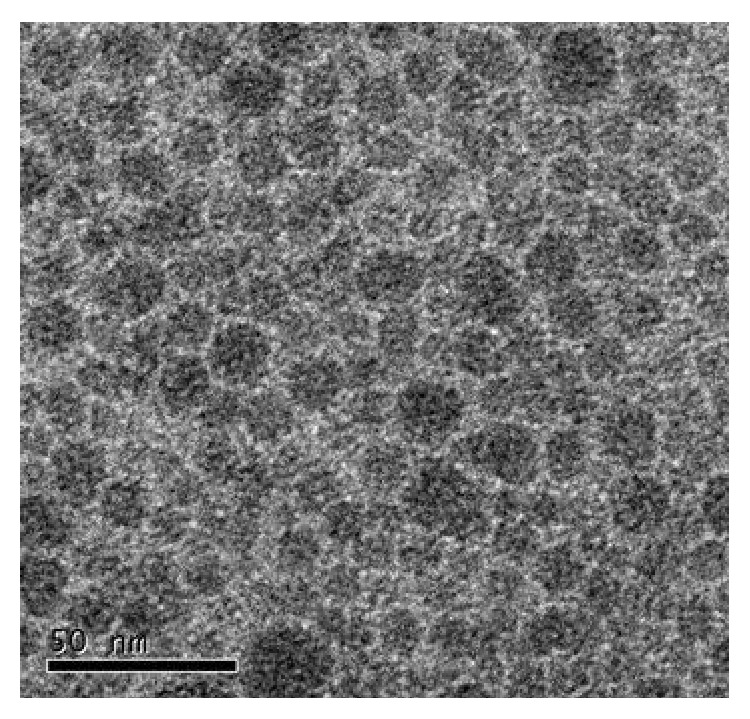
TEM image of nanosilica nanoparticles, the scale was shown in 50 nm.

**Figure 2:**
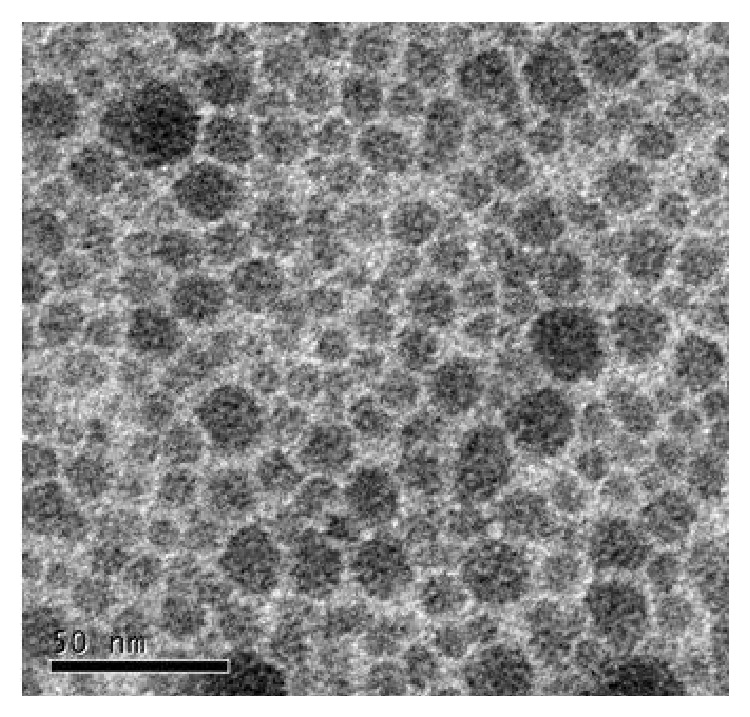
TEM image of polyacrylate/nanosilica nanoparticles, the scale was shown in 50 nm.

To characterize the nanoparticles, two kinds of particle emulsions (nanosilica and polyacrylate/nanosilica) were analyzed by TEM. As shown in Figures 1 and 2, the particle sizes of both nanomaterials ranged from 15 to 25 nm, consistent with the company description as 20 nm average diameters of the particles.

### 3.2. Mortality and General Condition of Animals

There were no observed increase in mortality among 0.9% saline-treated animals and no obvious abnormalities. However, a 30% mortality rate (3 out of 10 in each exposure group during the entire observation time) was observed in all nanosilica, polyacrylate/nanosilica exposed groups 24 hours after instillation. Autopsies showed significant pneumonedema and stethemia. All the animals in those groups displayed a shortness of breath, abated mobility, prolonged reaction time, and reduced food intake.

### 3.3. Impaired Respiratory Function in Exposed Animals

Upon 24 hrs after the exposure, as shown in Table 1 when compared to saline controls, pH decreased in animals from both exposure groups, but the differences were not significant (*P* > 0.05). Additionally, PCO_2_ was increased (no significant differences, *P* > 0.05) while PO_2_ significantly decreased in both exposure groups compared to controls (*P* < 0.05), suggesting an impaired respiratory function in nanoparticle exposed animals. Differences of PO_2_ within the two exposure groups were not found to be statistically different from each other (*P* > 0.05), indicating comparable respiratory toxicities in these two kinds of nanoparticles. We proceed with the observation time to 72 hrs, as shown in Table 2; blood gas analysis 72 hrs. after exposure is comparable to the 24 hr. group. Compared with control animals, pH decreased but was not significant (*P* > 0.05). PCO_2_ was also elevated (not significant *P* > 0.05) and PO_2_ was found to be significantly reduced (*P* < 0.05). There was no significant difference in PO_2_ within the exposure groups (*P* > 0.05), which were collectively similar to the blood gas results we found in animals 24 hrs. after exposure.

### 3.4. Reduced WBC in Exposed Animals

As shown in Table 3 WBC and monocyte count decreased in exposed animals compared to controls, but the differences were not found to be significant (*P* > 0.05). It was found that neutrophil count was significantly reduced (*P* < 0.05) due to exposure when compared to controls. There was no significant difference in neutrophil count found between the two exposure groups (*P* > 0.05). With a further look at the blood cell counts of animals 72 hrs after exposure (Table 4) WBC, neutrophil, and monocytes decreased in exposed animals when compared with control animals; however, the differences were not found to be statistically significant (*P* > 0.05). Eosinophils were analyzed at the same time with WBC; however, no statistically significant change was found in eosinophils.

### 3.5. Liver Injury in Exposed Animals

To examine whether the liver functions were affected by nanoparticle exposure, serum ALT and LDH were used as well accepted biomarkers for liver damage. As shown in Table 5, exposed groups showed significantly increased ALT and LDH (*P* < 0.05) compared to controls. No significant differences were observed within exposed groups in ALT or LDH (*P* > 0.05). Seventy-two hours after exposure animals exposed to nanosilica or polyacrylate/nanosilica exhibited slight increases in ALT but the differences were not significant (Table 6, *P* > 0.05). LDH levels in exposed animals significantly increased (*P* < 0.05) compared to saline controls. The differences in the LDH levels within the two exposure groups were not statistically significant (*P* > 0.05).

### 3.6. Pleural Effusion and Pericardial Effusion in Exposed Animals

**Figure 3:**
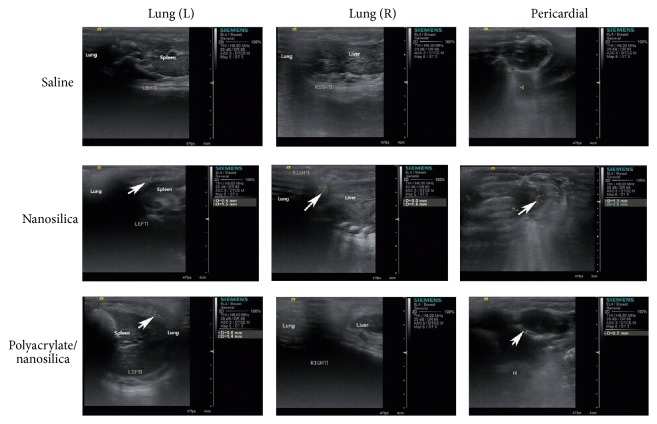
Ultrasound images of rats 120 hrs after exposure. L: left, R: right. The treatments were shown in left column and the organs subject to ultrasound examination were shown on the top row. White arrows showed the area of effusion observed under ultrasound.

Ultrasound imaging was performed 120 hrs. after intratracheal instillation to observe pleural effusion and pericardial effusion as a marker of pulmonary injury. No pleural effusion was observed in saline-treated animals (Figure 3) but was observed in animals exposed to nanosilica (Figure 3). In animals exposed to polyacrylate/nanosilica, pleural effusion was observed in the left lung, but not in the right (Figure 3). In addition, pericardial effusion was not observed in control animals (Figure 3) but was present in both exposure groups (Figure 3).

## 4. Discussion

The wide applications of nanotechnology have increased the chance of human exposure to nanoparticles in their daily lives. Previous studies have demonstrated that nanoparticles can cause health effects systemically; both molecular and cellular levels were examined [23–25]. Silicon oxide nanoparticle families have been shown to be an important particle in industrial use but have also been shown to induce oxidative stress lipid peroxidation and cause damage to tissues and cells [10–15, 26]. As a new material in this family, polyacrylate/nanosilica is becoming more widely used and was assumed to be a safe material in occupational settings [27]. However, previous research by our group showed increases in mortality in exposed workers, while pleural effusions, pulmonary fibrosis, and hypoxaemia were found in those workers exposed to polyacrylate/nanosilica [22]. In the current study, the toxicity of polyacrylate/nanosilica and nanosilica was compared in multiple organs and it was found that the two substances elicited similar multisystem toxicity in animals.

Several epidemiologic studies have found associations of ambient nanosized particles with adverse respiratory and cardiovascular effects resulting in morbidity and mortality in susceptible parts of the population [28–32]. Our results suggest that, compared to controls, exposure to nanosilica and polyacrylate/nanosilica resulted in a higher mortality, a shortness of breath, a weakened mobility, and a reduced feed quantity, which can be explained by irritation from pleural effusion and pericardial effusion in exposed animals. The results of the ultrasound imaging indicated that the exposure of nanosilica or polyacrylate/nanosilica was more likely to cause pleural effusion and pericardial effusion. This is also consistent with previous clinical observations in workers exposed to polyacrylate/nanosilica [22], indicating nanosilica and polyacrylate/nanosilica nanoparticles exposed rats exhibited similar consequences as humans exposed to those particles.

Studies in animals using laboratory-generated model nanoparticles or ambient nanosized particles showed that nanosized particles consistently induced pulmonary inflammatory responses and lung fibrogenesis in animals [13, 33–35]. In our research within 24 and 72 hours after exposure, arterial blood gas analysis showed that exposure to nanosilica and polyacrylate/nanosilica led to a significant drop in arterial PO_2_, as well as an increasing trend in PCO_2_ compared to controls. This was in line with the results of the clinical trial analysis of workers subjected to polyacrylate/nanosilica previously reported by our group [22]. With respect to nanosilica-triggered lung toxicity reported by Ying et al. [26], nanosilica yielded lung toxicity, similar to polyacrylate/nanosilica.

In this study routine blood testing and blood biochemical assay of ALT and LDH were conducted at 24 and 72 hr. after exposure. The results of this study showed that exposure to nanosilica, or polyacrylate/nanosilica, resulted in a downward trend in WBC, neutrophils, and monocyte counts, as well as a significant fall in neutrophils; this was in line with the observations we made in workers subjected to polyacrylate/nanosilica exposures [22]. Implying that nanosilica and polyacrylate/nanosilica could exert a similar toxic effect, in human and in rats, the molecular mechanisms remain far from clear and more researches are warranted. In addition, compared to controls, ALT and LDH presented an upward trend in exposure groups, and both ALT and LDH rose markedly, indicating the existence of liver damage after the exposures. The results indicate that nanosilica and polyacrylate/nanosilica have a similar toxicity, which is consistent with the increase of ALT in workers exposed to polyacrylate/nanosilica previously reported [22]. The liver is an important organ for detoxification in the body and plays a vital role in the metabolism of nanomaterials, specifically nanosilica. It has been shown that tail vein injection of nanosilica resulted in significant accumulation of nanoparticles in the liver and resulted in liver injury [14, 36]. Previous study showed an increased incidence of liver fibrosis after 84 days of nanostructured silica exposure [15]. Oxidative stress in mouse livers was notably prevalent after exposure to nanosilica, resulting in hepatic injury [37, 38]. The results of serum ALT and LDH levels in the exposed animals in our study supported the liver damaging of silica containing nanoparticles.

Polyacrylate is the homopolymer or copolymer of acrylate. After synthesis with nanosilica, this novel material has come into widespread use. Polyacrylate/nanosilica has a similar toxicity to nanosilica, as clinical trials and* in vivo* experimentation have shown. This study shows that this material can cause multiple organ toxicity, similar to nanosilica, which should raise the level of concern when handling this material. The research on molecular mechanism and toxicity of polyacrylate/nanosilica is warranted to secure the safe industrial application of these nanoparticles.

## Figures and Tables

**Table 1:** pH, PO_2_, and PCO_2_ levels in artery blood of rats in 24 hrs. after exposure (*N* = 6, $\overset{-}{x}\pm s$).

Exposure group	pH	PO_2_ (mmHg)	PCO_2_ (mmHg)
Saline	7.34 ± 0.04	85.83 ± 6.88	47.33 ± 4.36
Nanosilica	7.32 ± 0.06^#^	65.17 ± 16.43^▲^	50.67 ± 5.31^#^
Polyacrylate/nanosilica	7.32 ± 0.05^#^	66.17 ± 18.09^▲*∗*^	50.00 ± 7.66^#^

^#^ *P* > 0.05 when compared with saline group; ^▲^ *P* < 0.05 when compared with saline group; ^*∗*^ *P* > 0.05 when compared with nanosilica group.

**Table 2:** pH, PO_2_, and PCO_2_ levels in artery blood of rats in 72 hrs. after exposure (*N* = 6, $\overset{-}{x}\pm s$).

Exposure group	pH	PO_2_ (mmHg)	PCO_2_ (mmHg)
Saline	7.34 ± 0.04	85.33 ± 8.64	47.50 ± 7.39
Nanosilica	7.32 ± 0.03^#^	66.00 ± 16.02^▲^	49.50 ± 7.00^#^
Polyacrylate/nanosilica	7.32 ± 0.05^#^	66.67 ± 15.70^▲*∗*^	49.17 ± 8.51^#^

^#^ *P* > 0.05 when compared with saline group; ^▲^ *P* < 0.05 when compared with saline group; ^*∗*^ *P* > 0.05 when compared with nanosilica group.

**Table 3:** WBC, neutrophil, and monocyte count 24 hrs. after exposure (*N* = 6, $\overset{-}{x}\pm s$).

Exposure group	WBC × 10^9^/L	Neutrophil × 10^9^/L	Monocyte × 10^9^/L
Saline	3.46 ± 0.68	0.84 ± 0.07	0.11 ± 0.03
Nanosilica	3.07 ± 0.77^#^	0.49 ± 0.06^▲^	0.06 ± 0.01^#^
Polyacrylate/nanosilica	3.18 ± 1.23^#^	0.44 ± 0.05^▲*∗*^	0.06 ± 0.01^#^

^#^ *P* > 0.05 when compared with saline group; ^▲^ *P* < 0.05 when compared with saline group; ^*∗*^ *P* > 0.05 when compared with nanosilica group.

**Table 4:** WBC, neutrophil, and monocyte count 72 hrs. after exposure (*N* = 6, $\overset{-}{x}\pm s$).

Exposure group	WBC × 10^9^/L	Neutrophil × 10^9^/L	Monocyte × 10^9^/L
Saline	3.61 ± 0.82	0.95 ± 0.11	0.08 ± 0.02
Nanosilica	2.79 ± 0.49^#^	0.82 ± 0.19^#^	0.06 ± 0.01^#^
Polyacrylate/nanosilica	2.62 ± 0.66^#^	0.78 ± 0.11^#^	0.04 ± 0.01^#^

^#^ *P* > 0.05 when compared with saline group.

**Table 5:** ALT and LDH levels 24 hrs. after exposure (*N* = 6, $\overset{-}{x}\pm s$).

Exposure group	ALT (U/L)	LDH (U/L)
Saline	39.17 ± 8.70	159.83 ± 15.04
Nanosilica	51.67 ± 8.45^▲^	303.00 ± 12.04^▲^
Polyacrylate/nanosilica	50.33 ± 4.88^▲*∗*^	311.17 ± 17.89^▲*∗*^

^▲^ *P* < 0.05 when compared with saline group; ^*∗*^ *P* > 0.05 when compared with nanosilica group.

**Table 6:** ALT and LDH levels 72 hrs. after exposure (*N* = 6, $\overset{-}{x}\pm s$).

Exposure group	ALT (U/L)	LDH (U/L)
Saline	40.00 ± 5.06	167.50 ± 15.88
Nanosilica	44.67 ± 3.07^#^	330.83 ± 15.56^▲^
Polyacrylate/nanosilica	45.33 ± 4.45^#^	364.50 ± 14.07^▲*∗*^

^#^ *P* > 0.05 when compared with saline group; ^▲^ *P* < 0.05 when compared with saline group; ^*∗*^ *P* > 0.05 when compared with nanosilica group.
